# Artificial sleep-like up/down-states induce synaptic plasticity in cortical neurons from mouse brain slices

**DOI:** 10.3389/fncel.2022.948327

**Published:** 2022-10-14

**Authors:** Gai-Linn Kay Besing, Emily Kate St. John, Cobie Victoria Potesta, Martin J. Gallagher, Chengwen Zhou

**Affiliations:** ^1^Departments of Neurology, Vanderbilt University Medical Center, Nashville, TN, United States; ^2^Neuroscience Graduate Program, Vanderbilt University Medical Center, Nashville, TN, United States

**Keywords:** homeostatic synaptic plasticity, brain slices, sleep, up/down-state, whole-cell recordings

## Abstract

During non-rapid eye movement (NREM) sleep, cortical neuron activity alternates between a depolarized (firing, up-state) and a hyperpolarized state (down-state) coinciding with delta electroencephalogram (EEG) slow-wave oscillation (SWO, 0. 5–4 Hz) *in vivo*. Recently, we have found that artificial sleep-like up/down-states can potentiate synaptic strength in layer V cortical neurons *ex vivo*. Using mouse coronal brain slices, whole cell voltage-clamp recordings were made from layer V cortical pyramidal neurons to record spontaneous excitatory synaptic currents (sEPSCs) and inhibitory synaptic currents (sIPSCs). Artificial sleep-like up/down-states (as SWOs, 0.5 Hz, 10 min, current clamp mode) were induced by injecting sinusoidal currents into layer V cortical neurons. Baseline pre-SWO recordings were recorded for 5 min and post-SWO recordings for at least 25–30 min. Compared to pre-SWO sEPSCs or sIPSCs, post-SWO sEPSCs or sIPSCs in layer V cortical neurons exhibited significantly larger amplitudes and a higher frequency for 30 min. This finding suggests that both sEPSCs and sIPSCs could be potentiated in layer V cortical neurons by the low-level activity of SWOs, and sEPSCs and sIPSCs maintained a balance in layer V cortical neurons during pre- and post-SWO periods. Overall, this study presents an *ex vivo* method to show SWO's ability to induce synaptic plasticity in layer V cortical neurons, which may underlie sleep-related synaptic potentiation for sleep-related memory consolidation *in vivo*.

## Introduction

Non-rapid eye movement (NREM) sleep is when memories consolidate in the brain (Andrillon et al., 2011) while brain electroencephalogram (EEG) activity exhibits delta frequency (0.5–4 Hz), large amplitude oscillations with a high incidence of sleep spindles (Cantero et al., 2003; Andrillon et al., 2011). Meanwhile, cortical neurons go through alternating silent hyperpolarized states (down-state) and depolarized states (up-state, with action potential firing) (Shu et al., 2003; Saper et al., 2005; Hengen et al., 2016; Watson et al., 2016; Levenstein et al., 2017, 2019). Several *ex vivo* and *in vivo* methods such as tetanus stimuli, theta burst, and delta waves have been used to induce synaptic potentiation in cortical neurons (Petersen et al., 2003; Chauvette et al., 2012; Abrahamsson et al., 2016; Kouvaros and Papatheodoropoulos, 2016; Rodrigues et al., 2021). However, the activity patterns in these methods to induce synaptic potentiation seem to be more artificial compared to the cortical neuronal up/down-state activity patterns [as slow-wave oscillations (SWOs)] in the brain during NREM sleep. Moreover, a modified artificial cerebrospinal fluid (ACSF) has been used to induce the up/down-state pattern in neurons within brain slices (Hajos and Mody, 2009). However, we still do not know whether this up/down-state pattern can induce synaptic plasticity in cortical neurons or not. Only in work (Kurotani et al., 2008), a 0.5-Hz alternate up/down activity pattern has been used to induce somatic GABAergic inhibitory, but non-excitatory, synaptic potentiation in layer V cortical neurons. Here, we use one sinusoidal current injection within delta frequency (0.5 Hz alternate up/down-states, a low-level activity state) (Zhang et al., 2021) to induce both excitatory and inhibitory synaptic potentiation in layer V cortical neurons within mouse brain slice preparations *ex vivo*, which share intracellular events with synaptic plasticity in cortical neurons (Turrigiano and Nelson, 2004; Turrigiano, 2008).

## Materials and equipment

All chemicals (Table 1) were purchased from Millpore Sigma, Inc. (Burlington, MA, USA) (original as Sigma-Aldrich) unless otherwise stated.

(a) Dissection solution (mM): 214 sucrose, 2.5 KCl, 1.25 NaH_2_PO_4_, 0.5 CaCl_2_, 10 MgSO_4_, 24 NaHCO_3_, and 11 D-glucose;
(b) ACSF components (mM): 126 NaCl, 2.5 KCl, 1.25 NaH_2_PO_4_, 2 CaCl_2_, 2 MgCl_2_, 26 NaHCO_3_, and 10 D-glucose (pH 7.4);
(c) Intracellular solution components for spontaneous excitatory synaptic current (sEPSC) recordings (mM): 120 K-gluconate, 11 KCl, 1 MgCl_2_, 1 CaCl_2_, 0.6 EGTA, 10 HEPES, 2 Na-ATP, 0.6 Na-GTP, 10 K-creatine-phosphate, and pH 7.3 (Huguenard and Prince, 1994; Schofield et al., 2009) (Cl^−^ reversal potential −55.8 mV);
(d) Intracellular solution components for sIPSC recordings (mM): 65 K-gluconate, 65 KCl, 10 NaCl, 5 MgSO_4_, 0.6 EGTA, 10 HEPES, 2 Na-ATP, 0.6 Na-GTP, 10 K-creatine-phosphate, and pH 7.3 (Kurotani et al., 2008) (Cl^−^ reversal potential −15 mV);

**Table 1:** Chemical list for this method study.

**Chemical #**	**Suppliers**	**Catalog #**
NaCl	Sigma-Aldrich	S9888-1KG
KCl	Sigma-Aldrich	P3911-500G
NaH_2_PO_4_	Sigma-Aldrich	S0751-100G
${\text{CaCl}}_{2}^{.}$2H_2_O	Sigma-Aldrich	C8106-500G
${\text{MgCl}}_{2}^{.}$6H_2_O	Sigma-Aldrich	208337-100G
NaHCO_3_	Sigma-Aldrich	S5761-500G
MgSO_4_	Sigma-Aldrich	M2643-500G
Sucrose	Sigma-Aldrich	S0398-1KG
D-glucose	Sigma-Aldrich	G8270-100G
K-gluconate	Sigma-Aldrich	P1847-100G
EGTA	Sigma-Aldrich	E3889-100G
HEPES	Sigma-Aldrich	H3375-100G
Na-ATP	Sigma-Aldrich	A2383-1G
Na-GTP	Sigma-Aldrich	51120-25mg
K-creatine-phosphate	Calbiochem	237911-250mg
NBQX	TOCRIS	0373-10 mg

95%O_2_/5%CO_2_ from A to L compressed gases company and Isoflurane (NDC66794-017-25, Piramal Critical Care, Inc., Bethlehem, PA, USA);

26G Needles (BD, Inc., Franklin Lakes, NJ, USA) and Loctite Superglue (Henkel Corp., Rocky Hill, CT, USA).

### Equipment

Leica Vibrotome VT1200S, Nikon infrared/differential interference contrast microscope (IR-DIC) (Eclipse FN1, Nikon Corp., Inc., Melville, NY, USA) with Q-imaging system, Micromanipulator MPC-200 (Sutter Instrument, Novato, CA, USA), incubation chamber, TC-344B Dual automatic temperature controller (Warner Instrument, Inc.), PP-830 electrode puller (Narishige, Japan), glass capillaries (PG10165-4, 1.6 mm OD) (WPI, Inc., Sarasota, FL, USA), MultiClamp 700B amplifier, One Digidata 1440A, and Clampex 10/Clampfit 10 software (Molecular Devices, Inc., Union City, CA, USA).

## Methods

### The following number in parentheses indicates the sequence of the whole method

#### Mouse brain slice preparation

All procedures were in accordance with the guidelines set by the Institutional Animal Care and Use Committee of Vanderbilt University Medical Center as before (Zhou et al., 2012, 2013; Zhang et al., 2021) (a) Mice aged P60-150 (both male and female) were used in this study. Mice were deeply anesthetized with 5% isoflurane [inhaled, 3–5 min], and the mouse chest wall was cut to expose the heart for a left ventricle cardiac perfusion with an ice-cold dissection solution (injected with 26G needles) after the right atrium was cut. While fluids flowing out from the right atrium changed from a red blood color to a clear solution, and mice were then decapitated and brains were taken out to put into ice-cooled dissection solution with 95%O_2_/5%CO_2_ bubbled. This step would take 2–3 min. (b) Mouse brain tissues were then mounted on the cutting plate with a superglue and placed into the vibratome section chamber (containing an ice-cold dissection solution at 4°C). Mouse coronal brain slices (320 μm thickness) containing the somatosensory cortex were prepared with a vibratome (Leica VT 1200S, Leica Biosystems, Inc.). This step would take 5–10 min to finish before mouse brain slices were transferred to and incubated in a glass chamber (containing continuously oxygenated ACSF), bathed in 35–36°C water for 40 min. (c) Finally, the incubation chamber (with mouse brain slices) was taken out to remain at room temperature for at least 1 h before electrophysiological recordings at 32°C.

#### Whole-cell recordings of layer V cortical neurons within mouse brain slices

**Figure 1:**
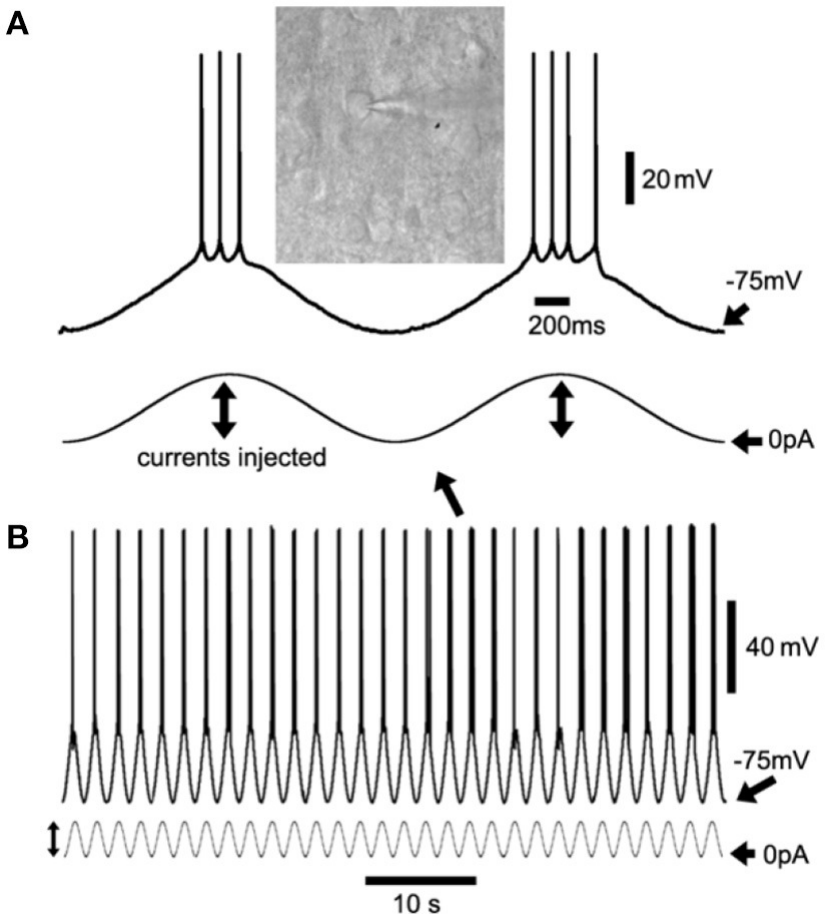
Sinusoidal current injection to induce cortical neuron up/down-states as slow-wave oscillations (SWOs). **(A)** Shows the two consecutive up/down-states with action potentials riding at the peaks with an inset of differential interference contrast (DIC) layer V cortical neuron image, expanded from two consecutive up/down-states in **(B)** (with an arrow indicating). **(B)** Shows the 60-s episode of SWOs (0.5 Hz) with injected sinusoidal current waveforms to induce neuronal up/down-states as SWOs. Scale bars are indicated as labeled. The vertical lines with dual arrows in **(A,B)** indicate the injected sinusoidal current amplitudes for up- (to induce action potentials) and down-states (0 pA injection, unless stated in Sections “Method/Result”). The sinusoidal waves are not in the same scale in **(A,B)**.

(a) After ACSF at 32°C was released through the recording system for 5 min (flow speed 1–1.5 ml/min), one mouse brain slice containing the somatosensory cortex was transferred and kept in a recording chamber with a slice grid (WPI, Inc., Sarasota, FL, USA). (b) Under a Nikon IR-DIC microscope (Eclipse FN1, Nikon Corp., Inc., Melville, NY, USA), layer V cortical pyramidal neurons within the somatosensory cortex were identified by their large apical dendrites and large cell somas (Figure 1, inset). This step would take 2–3 min. (c) One electrode was made through an electrode puller and filled with an intracellular solution before use. Be cautious to avoid air bubbles at the electrode tip. Then, the filled electrode was loaded onto an electrode holder of one patch-clamp headstage (Molecular Devices, Inc.) with a positive air pressure inside. Subsequently, the electrode tip was lowered (through a micromanipulator) into the perfusion ACSF in the recording chamber. This step would take 5 min. (d) With the Clampex 10 (Molecular Devices, Inc., Union City, CA, USA) in the bath-test mode (5 mV commanding voltage, voltage-clamp mode), this filled electrode should have a resistance of 2–5 m*Ω* with one internal solution and liquid-junction potentials were zeroed. Then, the electrode tip was moved to one selected layer V cortical neuronal soma with a micromanipulator to establish the whole-cell recording mode (voltage-clamp mode) with a good/stable serial resistance (Ra) around ~12 m*Ω*. This step would take 5–10 min. (e) Different internal solutions were used to record spontaneous excitatory synaptic currents (sEPSCs) (Huguenard and Prince, 1994; Schofield et al., 2009) or inhibitory synaptic currents (sIPSCs). α*-amino-3-hydroxy-5-methyl-4-isoxazolepropionic acid receptor*- (AMPAR-) mediated sEPSCs were recorded at a holding potential of −55.8 mV (Cl^−^ reversal potential) with the ACSF. GABAergic receptor-mediated sIPSCs were recorded at a holding potential of −60 mV with AMPAR/kainate receptors being blocked (20 μm NBQX in the ACSF) (Kurotani et al., 2008). *IPSCs were recorded as inward currents at* –*60 mV with the corresponding sIPSC internal solution (Cl*^−^ *reversal potential at* –*15 mV)*. All pre-SWO baseline recordings would take at least 5 min with a stable Ra (<25 m*Ω* and no more than 20% changes). Then neuronal recordings would switch to the current-clamp mode for resting membrane potential measurement. Resting membrane potentials (down-state) should remain <-70 mV with occasional variances (depending on the electrode-cell sealing quality during recordings) or with a few spontaneous action potential firings. Occasionally, if resting membrane potential variances due to a few large synaptic potentials were above than −68 mV, we did inject steady negative currents to hold cell membrane potentials below −70 mV (down-state). Otherwise, this neuronal recording should be discarded and a new layer V cortical neuron would be tried with a new electrode filled with an internal solution. After stable resting membrane potentials were established with layer V cortical neurons, SWO induction would start for 10 min (see below). This step would take 5–20 min.

#### Artificial SWO (0.5 Hz) induction

(a) Artificial SWOs (0.5 Hz) were induced by injecting sinusoidal currents (200–300 pA at 0.5 Hz) (Figure 1B) into cells (the current-clamp mode) to forcefully cause neuronal membrane potential alterations between resting membrane potentials (troughs <-70 mV as down-state, 0 pA injection) and −45 mV (peaks as up-state) for 10 min. Injected current amplitudes (Figure 1B) were adjusted to generate 4–5 action potentials (APs) at the peak of sinusoidal currents. During SWO induction, action potentials' amplitudes should not show any significant decrease. Otherwise, it would suggest that the cell serial resistance Ra had significantly increased during recordings. If neuronal resting membrane potentials were depolarized when SWO induction was initiated, negative currents might be required to bring down the membrane potentials below −75 mV. Otherwise, the recordings would be discarded. This would take 10–15 min. (b) After 10 min of SWO induction in the current-clamp mode, neuronal recordings switched back to the voltage-clamp mode at −55.8 mV for sEPSCs recordings or −60 mV for sIPSCs for 30–40 min recording, depending on the types of intracellular solution used. Meanwhile, the Ra was continuously monitored during pre- and post-SWO recordings and recordings with a Ra larger than 25 m*Ω* or 20% change were discarded. This step would take 30–40 min. During the post-SWO sEPSC/sIPSC period, the caveats were to keep recordings long enough with the same stable Ra as that during pre-SWO period. Otherwise, it would be impossible to compare pre- and post-SWO sEPSC or sIPSC amplitudes, frequencies, and rising and decaying phases of synaptic events for the similar layer V cortical neurons. In addition, the addition of K-creatine-phosphate into an intracellular solution would decrease the run-down effect for long-time recordings, especially for high-temperature recordings (~32°C or above).

#### Data analysis

Data were collected by using one multiClamp 700B amplifier and Clampex 10 software (Molecular Devices, Inc., Union City, CA, USA), filtered at 2 kHz, and digitized at 20 kHz using the Digidata 1440A (Molecular Devices, Inc., Union City, CA, USA). (a) All consecutive pre- and post-SWO sEPSC or sIPSC sweeps (each sweep 5 s long) from the same neurons were concatenated as one file to be analyzed by using the same threshold settings for all synaptic event detections. All traces with large noises (>6–8 pA) were discarded if present. Both sEPSCs and sIPSCs were analyzed with a threshold detection method (2.5X baseline RMS, 5–6 pA) by using the Clampfit 10.0 software program (Schofield et al., 2009; Rakhade et al., 2012; Zhou et al., 2012; Zhang et al., 2021). All detected sEPSC and sIPSC events were visually confirmed to ensure that their rising and decaying waveforms had normal dynamics. This step would take 10–30 min to finish. (b) After this step, sEPSC and sIPSC histogram and cumulative distribution graphs were constructed with the Clampfit 10.0 software and a Kolmogorov–Smirnov (K–S) non-parametric test was performed with the same number of random synaptic events from pre- and post-SWO recordings. sEPSC or sIPSC amplitudes and frequencies were calculated for synaptic events during pre-SWO baseline 5 min or post-SWO at 25–30 min (for a 5-min duration). All figures were prepared with Microsoft Excel, SigmaPlot/Stat, and Adobe Photoshop software. Data were expressed as mean ± standard errors of mean (SEM). Two-way analysis of variance (ANOVA) and Holm–Sidak tests were used if required. This step would take 30–60 min.

## Results

### Artificial SWO induction in layer V cortical neurons

Using sinusoidal current injection in layer V cortical neurons, cortical neurons (Figure 1A, inset) were engaged into up/down-state alteration (0.5 Hz) (Figure 1), with hyperpolarization below −75 mV as down-states and depolarization as up-states accompanied by occasional action potential firing at the peaks. Artificially induced alternate up/down-states in layer V cortical neurons lasted for 10 min, which simulated neuronal up/down-states *in vivo* during NREM sleep (Petersen et al., 2003; Shu et al., 2003).

### Artificial SWO induction potentiates excitatory sEPSCs in layer V cortical neurons

**Figure 2:**
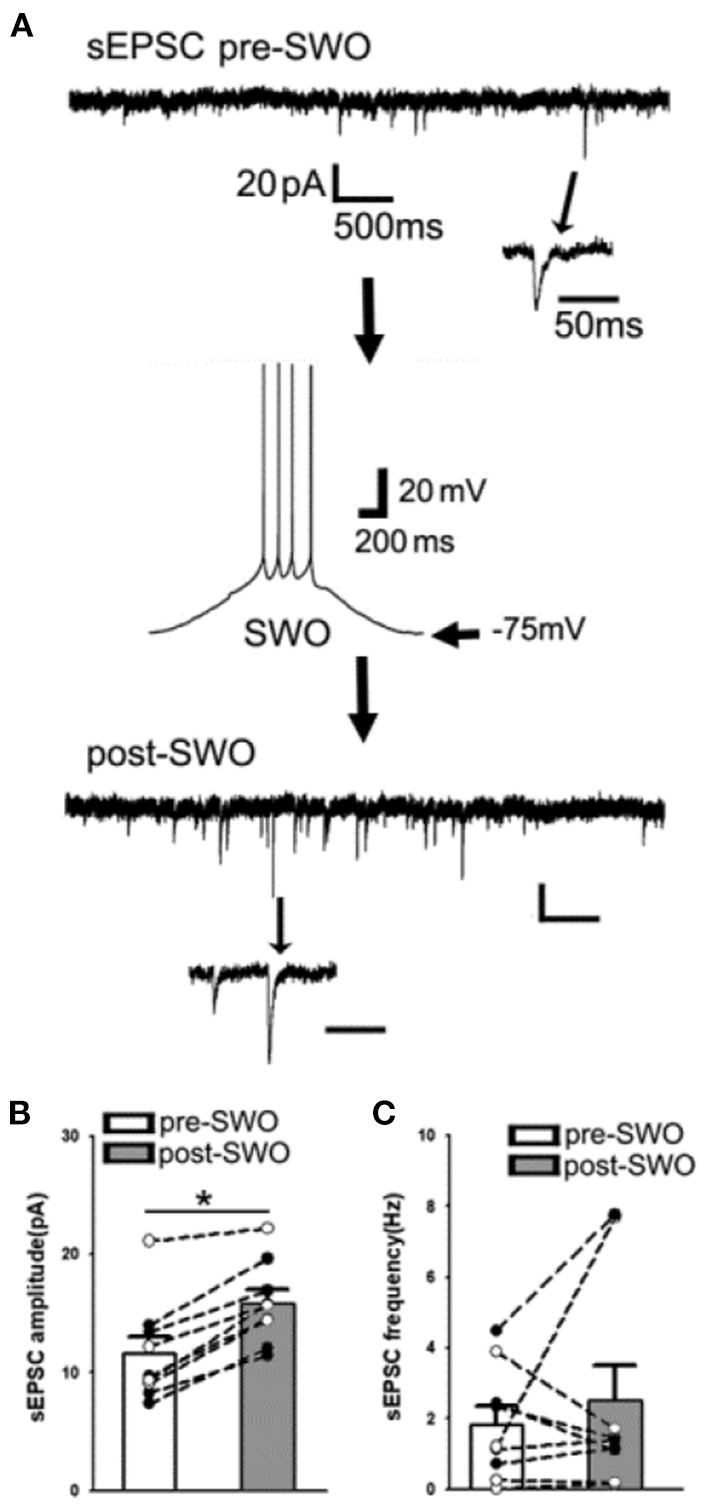
Artificial SWO induction potentiates spontaneous excitatory synaptic currents (sEPSCs) in layer V cortical neurons. **(A)** Shows one representative pre-SWO (top) and post-SWO sEPSC (below) in layer V cortical neurons and the middle panel shows one SWO. Individual sEPSC events (with arrow indicating) are expanded in a small temporal scale. Scale bars are indicated as labeled. **(B)** Shows the summary data of pre- (empty bar) and post-SWO (gray bar) sEPSC amplitudes from the same cortical neurons (dots connected with one line). **(C)** Shows the summary data of pre- (empty bar) and post-SWO (gray bar) sEPSC frequencies from the same cortical neurons (dots connected with one line). *Indicates a significant difference *p* < 0.05.

Compared to pre-SWO baseline sEPSCs in layer V cortical neurons, subsequent post-SWO sEPSC amplitudes in neurons increased and remained elevated for at least 30 min following SWO induction (Figure 2) [also Figure 1B in Zhang et al. (2021)]. Meanwhile, sEPSC amplitudes were significantly increased (Figures 2A,B) [*n* = 9 cells (seven mice), amplitude (pA), pre-SWO 11.60 ± 1.41 vs. post-SWO 15.85 ± 1.13 (at post-SWO 30 min), paired *t*-test *p* = 0.0005; frequency (Hz), pre-SWO 1.82 ± 0.52 vs. post-SWO 2.51 ± 1.00 (at post-SWO 30 min), paired *t*-test *p* = 0.459]. However, the rising and decaying phases of individual post-SWO sEPSC events did not show much changes, suggesting that similar AMPAR subunit composition (for decaying phase) might mediate the pre- and post-SWO sEPSCs in neurons.

### Artificial SWO induction potentiates inhibitory sIPSCs in layer V cortical neurons

**Figure 3:**
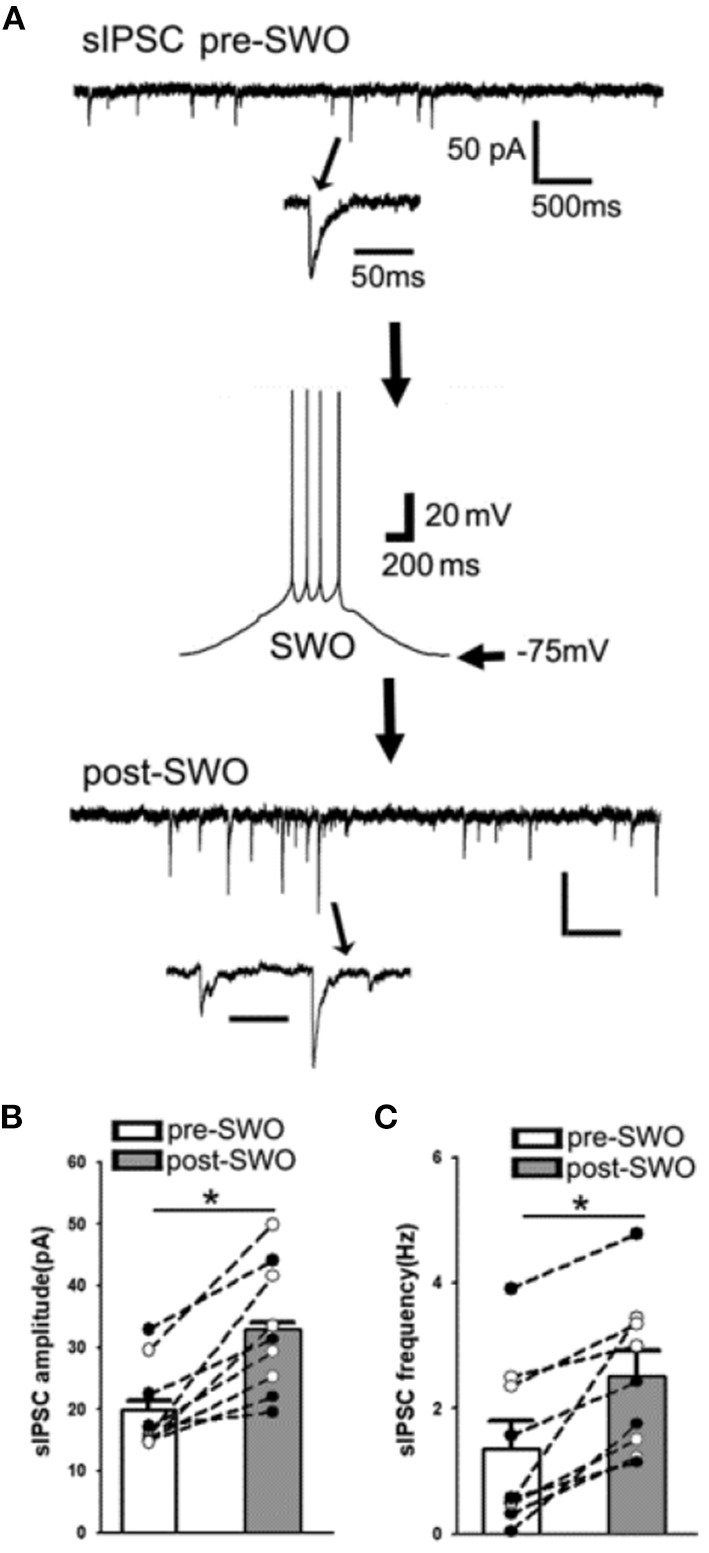
Artificial SWO induction potentiates spontaneous inhibitory synaptic currents (sIPSCs) in layer V cortical neurons. **(A)** One representative pre- (top) and post-SWO sIPSC (below) in layer V cortical neurons and the middle panel shows one SWO. Individual sIPSC events (with arrow indicating) are expanded in a small temporal scale. Scale bars are indicated as labeled. **(B)** Shows the summary data of pre- (empty bar) and post-SWO (gray bar) sIPSC amplitudes from the same cortical neurons (dots connected with one line). **(C)** Shows the summary data of pre- (empty bar) and post-SWO (gray bar) sIPSC frequencies from the same cortical neurons (dots connected with one line). *Indicates a significant difference *p* < 0.05.

Similar to post-SWO sEPSC potentiation, post-SWO sIPSCs also underwent similar potentiation following SWO induction in layer V cortical neurons. Post-SWO sIPSCs in cortical neurons exhibited a significant increase in both amplitudes and frequencies, compared to pre-SWO sIPSCs (Figures 3A–C) [*n* = 9 cells (seven mice), amplitude (pA), pre-SWO 19.96 ± 2.29 vs. post-SWO 32.95 ± 3.46 (at post-SWO 30 min), paired *t*-test *p* = 0.0007; frequency (Hz), pre-SWO 1.36 ± 0.43 vs. post-SWO 2.51 ± 0.40 (at post-SWO 30 min), paired *t*-test *p* = 0.002] [also Figure 3B in Zhang et al. (2021)]. Regarding its mechanism, this potentiation might depend on R-type calcium channels (Kurotani et al., 2008).

## Discussion

With the sinusoidal currents (0.5 Hz, alternate up/down-states as SWOs) and an *ex vivo* method, artificial SWOs were able to induce synaptic EPCS or IPSC potentiation in layer V cortical neurons *in vitro*. And, sEPSCs and sIPSCs might be balanced during the pre- and post-SWO period. The replicability of this straight-forward method has been shown in our previous manuscript (Zhang et al., 2021). Regarding the synaptic EPSC potentiation mechanism, the synaptic potentiation by SWOs depends on retinoid acid synthesis (Aoto et al., 2008) (which the N,N-diethylamino-benzaldehyde (DEAB) can block) and is further replicated by *in vivo* experiments (Catron et al., 2023), although it is a little different regarding DEAB purposes *in vivo*. In addition, the artificial SWO patterns (as low-level activity) are very similar to *in vivo* individual neuronal up/down-state patterns during NREM sleep. Compared to the low-level activity of SWOs during sleep, 50-Hz high-level activity could suppress sEPSCs in layer V cortical neurons [Supplementary Figure 2 in Zhang et al. (2021)], while 5-Hz high-level activity could attenuate sIPSCs in layer V cortical neurons [Supplementary Figure 3 in Zhang et al. (2021)]. These suggest that sEPSC and sIPSC may need different thresholds of high neuronal activity to trigger depression in cortical neurons (Turrigiano, 2008; Li et al., 2019).

Although SWO generation *in vivo* requires thalamocortical and other subcortical circuitry and neuromodulations, our simple method with artificial SWOs *in vitro* bypasses the complicated circuitries and neuromodulators and offers a surrogate way as SWOs *in vivo* to directly address its potential physiological effects on synapses or individual neurons. Previously, the modified ACSF (addition of 3.5 μm carbachol) has been used to induce neuronal up/down-states within brain slices (Reid et al., 1988; Sanchez-Vives and McCormick, 2000; Hajos and Mody, 2009; Neske et al., 2015). However, with this method, high-speed fast perfusion (at least 6–7 ml/min, also with double-side perfusion) of mouse brain slices is required. This fast perfusion makes it more difficult for long/stable recordings (at least 50 min) without losing good seals for recordings. Carbachol concentration needs to be low to avoid any seizure induction within neuronal networks (Frohlich et al., 2008; Cataldi et al., 2011; Hashimoto et al., 2017). In addition, with this method, not all neurons within mouse brain slices undergo a similar up/down alteration. However, we can induce synaptic potentiation with the fast perfusion method (Zhang et al., 2021) as our current method. In contrast, our method is simple and straightforward, allowing us to directly induce up/down-states in individual neurons without much influence on good seals for long, stable recordings.

Artificial alternating up/down-states by injection of sinusoidal currents (0.5 Hz) in layer V cortical neurons are very similar to intrinsic neuronal activity patterns *in vivo* during NREM sleep. Synaptic potentiation induced in layer V cortical neurons needs retinoid acid synthesis and low-level intracellular calcium depending on R-type calcium channels, but not L-type calcium channels, which permit larger calcium influx than R-type channels (Kurotani et al., 2008). In addition, suppression of synaptic EPSC potentiation can lead to the attenuation of spindle spindles *in vivo* based on an ongoing work. Thus, SWO activity patterns with this method may share some mechanisms with homeostatic synaptic plasticity (Turrigiano and Nelson, 2004; Turrigiano, 2008) for memory consolidation (Massimini et al., 2004; Andrillon et al., 2011). Although NREM sleep has been shown to scale down synaptic strength in the cortex during sleep (Diering et al., 2017; de Vivo et al., 2017), these works never directly show whether or not cortical neurons undergo high- or low-level activity (0.5 Hz SWOs), and whether these cortical neurons are the part of memory engram assembles after behavioral tests. Our current work does show that low-level activity in cortical neurons during NREM sleep (particularly 0.5 Hz SWOs) is able to potentiate synaptic strength in theory, which is compatible with memory consolidation during NREM sleep in the field of neuroscience.

Overall, our method offers a simple/replicable way to induce synaptic potentiation as another method of synaptic potentiation, with more intrinsic activity patterns compared to neuronal activity patterns *in vivo* during NREM sleep. This may mechanistically explain how NREM sleep activity engages synaptic potentiation in cortical neurons for memory consolidation in the brain.

## Data availability statement

The raw data supporting the conclusions of this article will be made available by the authors, without undue reservation.

## Ethics statement

The animal study was reviewed and approved by Vanderbilt University Medical Center Institutional Animal Care and Use Committee.

## Author contributions

CZ designed experiments. G-LB and CZ performed experiments. G-LB, ES, and CP provided animal care and surgery. G-LB, ES, and CZ analyzed data. G-LB and CZ wrote this manuscript and discussed it with MG. All authors contributed to the article and approved the submitted version.

## Funding

This work was supported by NIH Grants NINDS R01NS107424-01 (CZ) and NINDS/NIA R01 supplemental grant (CZ).

## Conflict of interest

The authors declare that the research was conducted in the absence of any commercial or financial relationships that could be construed as a potential conflict of interest.

## Publisher's note

All claims expressed in this article are solely those of the authors and do not necessarily represent those of their affiliated organizations, or those of the publisher, the editors and the reviewers. Any product that may be evaluated in this article, or claim that may be made by its manufacturer, is not guaranteed or endorsed by the publisher.
